# An Orbital Abscess Caused by Fusobacterium nucleatum Mimicking Tolosa-Hunt Syndrome

**DOI:** 10.7759/cureus.99130

**Published:** 2025-12-13

**Authors:** Tomohiro Shogase, Takuya Matsushita, Yuya Maeda, Yu Arakawa

**Affiliations:** 1 Department of Neurology, Kochi Medical School Hospital, Nankoku, JPN; 2 Department of Neurology, Kochi Medical School, Kochi University, Nankoku, JPN; 3 Department of Neurology, Kyushu University, Fukuoka, JPN; 4 Department of Ophthalmology, Kochi Medical School Hospital, Nankoku, JPN; 5 Department of Infectious Diseases, Kochi Medical School Hospital, Nankoku, JPN

**Keywords:** cranial nerve palsy, fusobacterium nucleatum, orbital abscess, tolosa-hunt syndrome, visual complications

## Abstract

*Fusobacterium* species, particularly *Fusobacterium nucleatum*, are anaerobic gram-negative bacteria found in the oral cavity and gastrointestinal tract. Infections caused by these organisms exhibit diverse clinical manifestations. Here we report a rare case of orbital abscess caused by *Fusobacterium nucleatum,* which was initially misdiagnosed as Tolosa-Hunt syndrome. The patient was controlled conservatively, and surgical intervention was withheld due to poor prognosis, diabetes, and logistical limitations. This case report highlights the need to consider infection even when imaging and laboratory tests are unremarkable, and to emphasize the importance of close follow-up of orbital abscesses due to its aggressive nature.

## Introduction

Orbital abscess represents a critical ophthalmologic emergency requiring prompt diagnosis and intervention to prevent complications such as visual loss or intracranial extension. In certain cases, however, its clinical presentation may resemble non-infectious inflammatory conditions such as Tolosa-Hunt syndrome (THS). They share overlapping clinical features such as periorbital pain, cranial nerve palsies, and imaging findings limited to orbital soft tissue changes, making early differentiation challenging.

*Fusobacterium* species were traditionally regarded as commensal organisms of the oral cavity, gastrointestinal tract, and female genital tract. However, they are increasingly recognized as opportunistic pathogens capable of causing severe infections. including abscess formation and, less commonly, orbital involvement [1,2]. Among them, *Fusobacterium nucleatum (F. nucleatum)* is less frequently associated with systemic diseases compared to *Fusobacterium necrophorum*, and its involvement in orbital infections has not been reported to date. Orbital cellulitis and abscess formation are ophthalmologic emergencies that require prompt diagnosis and treatment to prevent irreversible vision loss [3]. The clinical features of orbital infections, however, may overlap with non-infectious inflammatory syndromes such as THS, which present painful ophthalmoplegia and cranial nerve palsies. In early stages, radiological findings may be minimal, complicating the distinction between infectious and inflammatory etiologies [4]. In this report, we describe a case of orbital cellulitis caused by *F. nucleatum* that was initially misdiagnosed as THS. The absence of systemic signs of infection and the negative blood cultures further complicated the diagnostic process. This case underscores the importance of considering infectious etiologies even when initial clinical and imaging findings suggest an inflammatory syndrome.

## Case presentation

A 57-year-old Japanese man with a history of type 2 diabetes, hypertension, and dyslipidemia underwent extraction of his right upper tooth eight weeks prior to admission. He first experienced mild frontal and temporal headaches shortly before the dental procedure, but his headache intensified after the treatment. Five weeks before admission, he noticed persistent diplopia, followed by right ptosis, retroorbital pain, and numbness in the right forehead, leading him to seek neurosurgical consultation. His right eye movement was severely restricted in all directions, but non-contrast magnetic resonance imaging (MRI) of the brain was unremarkable. His initial physician prescribed oral prednisolone 0.6 mg/kg/day based on the diagnosis of THS. His retroorbital pain, however, progressively worsened after the oral prednisolone was started. Due to his impaired vision and obvious proptosis, he was referred to our hospital for further investigation and was hospitalized on the same day.

**Figure 1:**
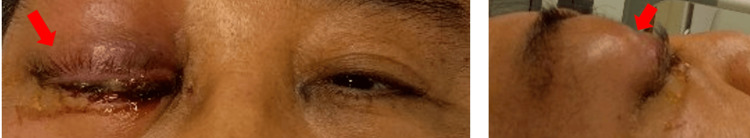
The appearance of the patient's eyes on admission As well as swollen right eyelid, marked protrusion of the right eye with large amounts of eye discharge was observed (arrow). The right eye movement was severely restricted in all directions.

**Figure 2:**
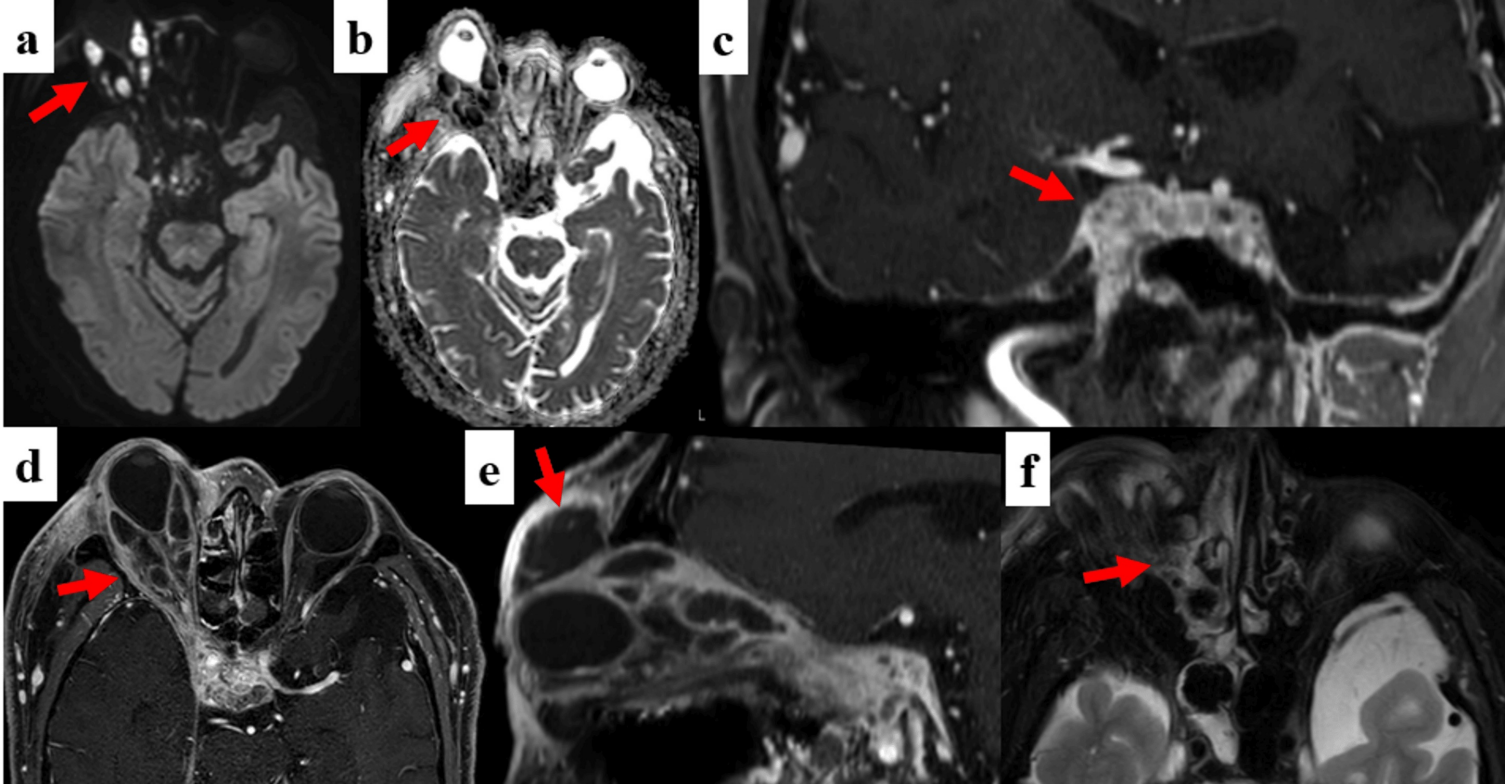
Orbital MRI on admission (a, b) Abscesses in extraocular muscles showed hyperintensity in diffusion-weighted imaging (DWI) with apparent diffusion coefficient (ADC) reduction (arrow). (c, d) Inflammation spread towards the right orbital apex and cavernous sinus (arrow), which showed prominent gadolinium enhancement. (e) There was also abscess formation in the right upper eyelid, where incisional drainage and sampling were done (arrow). (f) Fluid accumulation was seen in the right sphenoid sinus (arrow), suggesting sinusitis as one of the infection routes.

**Figure 3:**
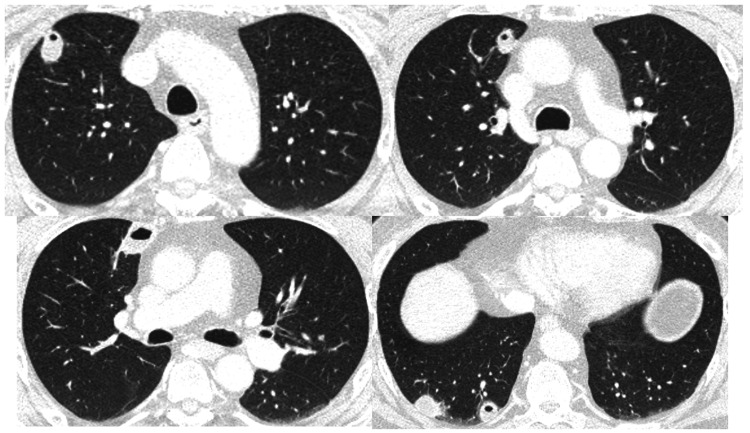
Chest CT on day two Multiple small abscesses were seen mainly in the patient's right lung. During the clinical course, a small amount of pleural effusion emerged in the right thoracic cavity.

On admission, he was awake and alert with normal vital signs. His right eyelid was so severely swollen that he couldn't open his right eye. His right eye had no light perception, and both the direct and indirect light reflexes of the affected eye were absent. Proptosis and conjunctival redness were also observed (Figure 1). Neurological examination revealed II (optic nerve), III (oculomotor nerve), IV (trochlear nerve), V1 (ophthalmic nerve), V2 (maxillary nerve), and VI (abducens nerve) palsies of the right cranial nerves, implying combined cavernous sinus syndrome and mechanical compression on the orbit. He also complained of his right-sided chest pain and coughing fit. Laboratory findings were notable for inflammatory changes (C-reactive protein 14.94 mg/dL, white blood cells 18,000/μL with neutrophil predominance), while two sets of blood culture were negative. Cerebrospinal fluid (CSF) was normocellular with no malignant cells, and CSF culture was negative. MRI on admission showed multiple abscesses in his right extraocular muscles, and inflammatory lesions extended from just behind his right eyeball to bilateral cavernous sinuses with prominent gadolinium enhancement (Figure 2). There was no evidence of meningitis or osteomyelitis, and computed tomographic angiography (CTA) showed no cerebrovascular involvement, including cavernous sinus thrombosis or carotid cavernous fistula. Contrast-enhanced CT scan showed multiple lung abscesses (Figure 3), but there were no signs of abscess formation in the deep neck and orofacial area.

**Figure 4:**
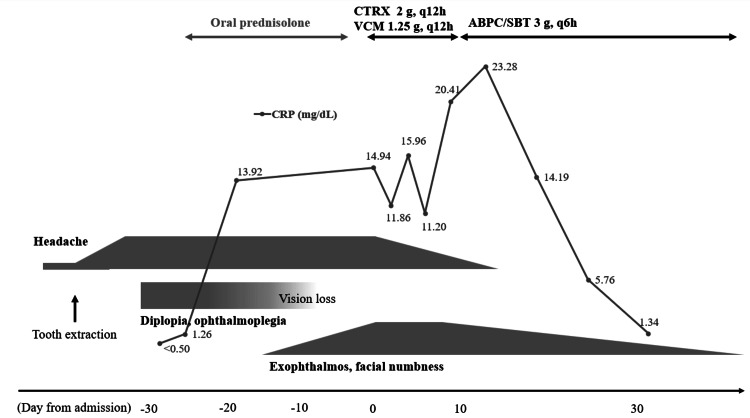
Clinical course Shortly after the patient got his tooth removed, he started to suffer from a headache, followed by impaired vision, diplopia, exophthalmos, and facial numbness. On his initial visit, the MRI was unremarkable, and the laboratory test showed no evident inflammation despite the presence of multiple cranial nerve palsies. Oral prednisolone backfired on disease control, and when follow-up MRI was performed, dozens of abscesses were looming out there. He was hospitalized immediately on the day of referral. Intravenous ampicillin sulbactam was quite effective, and we eventually managed the case conservatively. CRT - C-reactive protein; CTRX - ceftriaxone; VCM - vancomycin; ABPC/SBT - ampicillin/sulbactam

**Figure 5:**
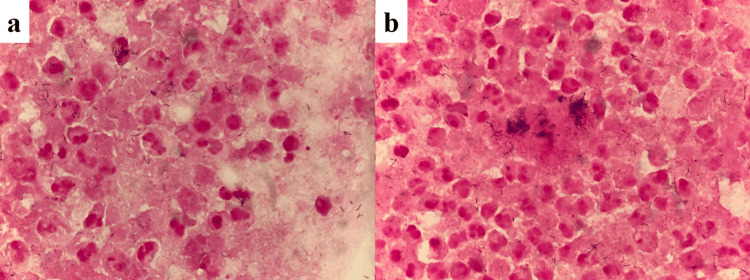
Sample of the pus in his right eyelid (a,b) Lots of Gram-variable-stained bacilli being phagocytosed were seen. They were later identified as *Fusobacterium nucleatum*. Both samples were Gram-stained (x1000 magnification).

The clinical course is shown in Figure 4. We started his treatment with intravenous ceftriaxone and vancomycin, but deformation of his right eyeball and optic nerve was already severe, leaving very little room for ocular function recovery. An incision and drainage of the upper eyelid was performed on day three to obtain purulent material for microbiological evaluation (Figure 5). Gram staining of the sample revealed numerous slender filamentous rods with variable staining characteristics, raising suspicion for an anaerobic infection. *F. nucleatum* was isolated from the culture; however, drug susceptibility testing could not be performed due to insufficient bacterial growth. Based on the culture result, antimicrobial therapy was switched to intravenous ampicillin-sulbactam.

Initially, there appeared to be minimal response to conservative treatment, and early surgical debridement was considered. However, after consultation with ophthalmologists, surgical intervention was deferred due to several factors: the patient had already lost light perception in the affected eye at the time of admission, indicating a poor visual prognosis; he also had poorly controlled diabetes, which was partially attributed to corticosteroid treatment, which posed a significant surgical risk. In addition, prompt surgery was not readily available at our institution. Fortunately, the postorbital abscesses began to regress spontaneously, and inflammatory markers improved over the following two weeks. Intravenous antibiotics were continued under close observation. 

The pulmonary abscesses also resolved gradually without invasive intervention. Although right-sided pleural effusion suggested associated pleuritis, it remained mild and did not require drainage. Chest pain also subsided gradually. A total of eight weeks of intravenous ampicillin-sulbactam was administered. Although visual function could not be restored, no recurrence of infection has been observed to date.

## Discussion

Retroorbital lesions often cause ocular pain and ophthalmoplegia, which is referred to as orbital apex syndrome. There is a broad range of differential diagnoses for orbital apex syndrome, from benign inflammatory diseases to life-threatening infections and malignant tumors. It often requires immediate investigation and treatment to prevent vision-threatening complications, particularly in cases demonstrating rapid aggravation of ocular symptoms. In our patient, we assume the primary infectious focus originated in the retroorbital region, which was probably triggered by recent dental treatment and sphenoid sinusitis. The infection subsequently extended to involve extraocular muscles and the cavernous sinus, and through the bloodstream, resulting in secondary pulmonary abscess formation. The absence of initial radiological findings strongly supports the hypothesis that there was pure unilateral cavernous sinus syndrome at first, and then mechanical compression by orbital abscess confined his eye movement. Although well-managed, his diabetes, combined with corticosteroid therapy, may have partially contributed to infectious susceptibility.

The bacteriology of orbital abscess varies depending on each clinical review. *Staphylococcus aureus*, *Staphylococcus epidermidis*, *Streptococci*, and *Haemophilus influenzae* are common causative bacteria, and anaerobes, including *Fusobacterium* species, are sometimes responsible for adult orbital abscess [3]. While *F. necrophorum* is more commonly reported in orbital abscesses [5] and septic thrombophlebitis of the internal jugular vein (Lemierre syndrome) [6], this is the first case implicating *F. nucleatum*. *F. nucleatum* infection is known to have male predominance and mainly to affect the middle-aged populations (over 40 years old) [2].

**Figure 6:**
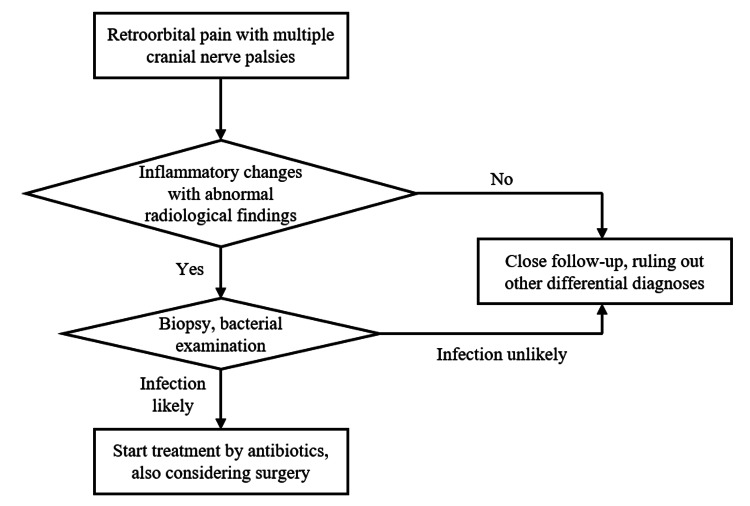
A simple algorithm for the management of painful ophthalmoplegia

THS, characterized by painful ophthalmoplegia and granulomatous inflammation of the cavernous sinus or orbital apex, typically demonstrates a dramatic response to corticosteroids and shows characteristic gadolinium enhancements on MRI. The frequency of MRI abnormalities in THS varies across studies. In a cohort of 31 patients, 64.5% showed abnormal MRI findings, including granulomatous inflammation of the cavernous sinus, superior orbital fissure involvement, or optic nerve lesions [7]. Reliance on imaging alone can be misleading, as early-stage abscesses, such as in our case, may evade detection on radiological screening, leading to diagnostic uncertainty. It is sometimes difficult for clinicians to distinguish infectious diseases from THS, and a previous report showed a case of invasive aspergillosis mimicking THS [8]. Notably, despite disseminated abscess formation across multiple organs, the blood culture was negative in our case. While previous case reports documented Lemierre syndrome [9] and large lung abscesses [10] caused by *F. nucleatum*, the blood cultures were positive in these cases. The annual incidence of *Fusobacterium* bacteremia is estimated to be 0.55/100,000, and several factors have been considered to raise the risk of the infection, such as malignancy, dialysis, chronic obstructive pulmonary disease, diabetes, and heart disease [1]. *Fusobacterium* species are difficult to isolate in blood cultures due to their susceptibility to oxygen [11], suggesting that sampling procedures and the quantity of bacteria in the bloodstream might have influenced our results. Despite the systemic, multiorgan infection implying bacteremia, his general appearance was not ill, and his vital signs were absolutely normal, suggesting that we must not rule out the early stage of orbital infection just because of negative blood culture and relatively good health state. This clinical presentation has not been described in previous reports and was possibly one of the reasons for diagnostic delay. We present a simple flow chart to illustrate how we can manage the case of painful ophthalmoplegia in Figure 6.

Given the irreversibility of vision loss at presentation, surgical intervention was considered unlikely to provide benefit and was therefore withheld in this case. A review from Saudi Arabia reports 38% of *Fusobacterium* infection cases developed one or more complications, in which thrombosis, endocarditis, and pulmonary embolism are relatively common [12]. Especially as to orbital abscess, quite a few reviews recommend surgical intervention in cases with larger abscess or optic nerve compression [13], poor response to conservative treatment in 48 hours [14,15], as well as the classical review by Harris suggesting urgent surgical drainage for orbital abscess [16]. When surgical intervention is not available, intensive monitoring, including serial orbital imaging and systemic assessments, is necessary to prevent further complications.

## Conclusions

We report a rare case of orbital abscess caused by *F. nucleatum*, initially misdiagnosed as THS due to isolated cranial nerve palsies and unremarkable initial imaging. The infection progressed to involve the cavernous sinus and lungs, forming multiple abscesses, despite negative blood cultures and the absence of systemic signs.

Intraorbital and cavernous sinus infection is one of the most crucial differential diagnoses for painful ophthalmoplegia or cavernous sinus syndrome of unknown origin. It often disguises itself as THS and other inflammatory diseases. Our case underscores the importance of considering anaerobic pathogens in culture-negative infections, particularly in patients with dental risk factors or immunocompromised status, showing normal radiological findings and negative blood culture are not necessarily sufficient in ruling out an early stage of infection. We should be more alert for signs of infection when there is little response to corticosteroids and the ocular symptom is getting worse. It necessitates urgent re-evaluation of initial diagnoses, which was also the key turning point in our case. In selected patients where surgery is infeasible or unlikely to alter the outcome, close observation and targeted antimicrobial therapy may offer a successful alternative.
